# Gender Representation at Turkish Anaesthesiology and Reanimation Congresses: Trends in Speakers, Session Chairs, and Society Leadership from 2015 to 2024

**DOI:** 10.4274/TJAR.2026.262473

**Published:** 2026-08-28

**Authors:** Sait Fatih Öner, Sevim Şenol Karataş

**Affiliations:** 1 University of Health Sciences Türkiye Elazığ Fethi Sekin City Hospital, Clinic of Anaesthesiology, Elazığ, Türkiye

**Keywords:** Anaesthesiology congresses, gender representation, invited speakers, session chairs, women in anaesthesiology

## Abstract

**Objective:**

This study evaluated gender representation among speakers, session chairs, and society leadership roles at the Turkish Society of Anaesthesiology and Reanimation (TARK) Congresses between 2015 and 2024, focusing on temporal patterns and subspecialty-based variation.

**Methods:**

This retrospective observational analysis of conference sessions included all scientific sessions listed in official TARK congress programmes from 2015 to 2024. Names, roles, and session topics were extracted from publicly available documents. In the absence of self-identified gender data, participants were categorised as women or men using names, academic titles, congress documents, and publicly accessible institutional or professional information, when clarification was required. Gender proportions were compared using chi-square tests. Temporal patterns were evaluated using logistic regression analyses, and subspecialty-specific analyses were performed using intensive care as the reference category. The leadership composition of the society was evaluated descriptively.

**Results:**

A total of 2,627 congress roles were analysed, including 966 session chairs and 1,661 speakers. Women accounted for 50.3% of session chairs and 50.8% of speakers, with no statistically significant temporal change during the study period. Marked variation was observed across subspecialties. Women were least represented in intensive care sessions (30.9%) and most represented in obstetric and paediatric anaesthesia sessions (82.1%), reflecting a higher relative representation of women speakers in the latter than in intensive care. Leadership composition varied across terms, with no persistent predominance of either gender according to the descriptive assessment.

**Conclusion:**

Gender representation at TARK congresses remained numerically balanced over the ten-year study period; however, the absence of a significant temporal increase and the presence of persistent subspecialty clustering suggest a stable but uneven distribution across academic areas.

**Main Points**

• Women accounted for approximately half of both speaker and session-chair roles at Turkish Society of Anaesthesiology and Reanimation Congresses between 2015 and 2024, with no significant temporal change in proportional representation.

• Gender representation varied markedly across subspecialties, with women representing 30.9% of speakers in intensive care and 82.1% in obstetric and paediatric anaesthesia.

• Compared with intensive care, women had substantially higher relative representation in obstetric/paediatric anaesthesia, neuroanaesthesia, and cardiothoracic anaesthesia.

• Overall numerical balance should not be interpreted as definitive evidence of equitable opportunity because year-matched workforce and society-membership denominators were unavailable.

## Introduction

Scientific meetings play a pivotal role in academic visibility, professional networking, and career advancement. Roles such as chairing sessions and giving invited talks are visible markers of academic recognition and may contribute to professional networking, academic visibility, and leadership trajectories. Beyond conferences, academic visibility and gender representation have also been examined using archival approaches in other scholarly contexts, such as authorship and editorial board composition in medical journals. For women academics in particular, visibility on scientific platforms represents an important component of professional development and sustained academic engagement. Nevertheless, accumulating evidence indicates that gender inequities persist in academic medicine and that these disparities are reflected in representation patterns at scientific conferences.^1, 2^

Despite the increasing proportion of women physicians in anaesthesiology, their representation as speakers and session chairs at scientific congresses has not consistently mirrored this growth. At the annual meetings of the American Society of Anesthesiologists (ASA), women have constituted only 22-25% of invited speakers, and additional imbalances have been reported in session chair roles.^3^ Similarly, analyses of the Canadian Anesthesiologists’ Society (CAS) meetings have shown that women accounted for merely 28.5% of invited speakers, with nearly half of sessions composed exclusively of men.^4^ These disparities appear even more pronounced across subspecialties. For example, women’s representation in cardiovascular anaesthesia sessions has been reported as low as 5% at certain meetings.^5^

Data from Asia reflect comparable trends. At the annual meetings of the Japanese Society of Anesthesiologists (JSA), women constituted only 17.9% of the speakers, despite representing approximately 40% of society members.^6^ Moreover, the finding that 44% of sessions consisted entirely of male speakers suggests gender-related barriers to accessing academic leadership and highlights potential structural imbalances in invitation and selection processes.

Programme committees, society leadership, and session coordinators play a critical role in constructing conference programmes. Prior studies have shown that sessions coordinated or chaired by women have significantly higher proportions of women speakers.^7^ This observation underscores the potential influence of gender diversity in programme coordination and session leadership on scientific visibility and representation patterns, and highlights the importance of inclusive approaches in congress programme planning.

In Türkiye, the Turkish Society of Anaesthesiology and Reanimation (TARK) Congress is one of the largest national scientific meetings in anaesthesiology and an important indicator of academic representation at the national level. However, to date, no study has systematically examined the long-term distribution of women and men among speakers and session chairs at TARK meetings. Given the steadily increasing proportion of women physicians in Türkiye, evaluating how women’s visibility is reflected in national scientific platforms is highly relevant to understanding patterns of academic representation and professional visibility.

Accordingly, this study aimed to examine gender representation among speakers and session chairs at the TARK congresses held between 2015 and 2024, with attention to session types, subspecialty areas, and society leadership structures. In addition to descriptive analyses, temporal patterns and subspecialty-based variation were analysed. In this respect, the present study constitutes one of the few long-term, data-driven analyses addressing representation patterns across speaking, chairing, and leadership roles in anaesthesiology congresses in Türkiye.

## Methods

### Ethics Statement

Ethical approval for this study was obtained from the Non-Interventional Research Ethics Committee of the University of Health Sciences Türkiye, Elazığ Fethi Sekin City Hospital (approval no: 2026/25-10, date: 05.03.2026). The study was conducted using publicly available congress programme data and did not involve patient records, clinical interventions, private personal health information, or direct contact with individuals. All analyses were performed at the aggregate level, and no individual-level judgements or evaluations were made.

### Study Design and Data Sources

This retrospective observational study evaluated the gender distribution of speakers and session chairs at national congresses organised by the TARK. The analysis covered congress programmes held between 2015 and 2024.

All scientific sessions, panels, and lectures listed in the official annual TARK congress programmes during the study period were included. Non-scientific events, social programme activities, opening and closing ceremonies without scientific presentations, industry-sponsored promotional sessions without identifiable academic speakers, and entries with insufficient role information were excluded from the analysis. Multiple appearances by the same individual in different roles or sessions were counted as separate congress roles because the unit of analysis was role-based representation, not unique individuals. Data were extracted from publicly available official congress programme documents. For each congress year, the following information was collected: the names of session chairs and speakers; session titles; presentation types; and the names of the TARK president and members of the organising committee. Programme documents were converted from PDF format to a standardised data-extraction form, and all entries were independently verified by a second investigator to ensure accuracy.

### Gender Classification and Subspecialty Grouping

In the absence of self-identified gender data in the congress programmes, participants were categorised as women or men using a structured, multi-step verification procedure. First, the participant names and academic titles listed in the official congress programmes were reviewed. For gender-neutral, uncommon, abbreviated, or otherwise uncertain names, the original congress PDF files were re-examined, and publicly accessible online sources were reviewed when available. These sources included institutional webpages, professional profiles, and publicly available photographs or biographical information. The final classification was made after cross-checking the sources and reaching agreement among the investigators.

This procedure was used only for aggregate-level analysis of gender representation and was not intended to define individual gender identity. Because self-identified gender information was not available, gender was analysed using a binary women/men framework, consistent with previous retrospective analyses of conference programmes. The potential for misclassification, particularly for gender-neutral names and participants with limited publicly available information, was considered a methodological limitation.

Sessions were grouped into major subspecialties based on session titles. When a session covered more than one topic, classification was based on the primary session theme. Subspecialty classifications were also reviewed for consistency, and uncertain classifications were resolved by consensus.

### Study Variables and Analytical Objectives

The primary study variables were congress year, participant role, gender category, session type, and subspecialty area. The primary outcomes were the annual proportions of women and men serving as session chairs and speakers at TARK congresses between 2015 and 2024. Secondary outcomes included temporal trends in gender representation, subspecialty-based variation among speakers, and the gender composition of TARK leadership, including society presidents and organising committee members.

Because the study used retrospective congress programme data, the analyses were designed to describe and compare representation patterns rather than test causal hypotheses. The analytical objectives were: (i) to determine the overall distribution of women and men among session chairs and speakers, (ii) to evaluate whether gender representation changed over the ten-year study period, (iii) to examine whether speaker representation differed across anaesthesiology subspecialties, and (iv) to describe the gender composition of society leadership during the corresponding period. Leadership data were analysed descriptively and not used to infer a causal relationship between leadership composition and the selection of speakers or session chairs.

### Statistical Analysis

Statistical analyses were performed using SPSS Statistics version 26.0 (IBM Corp., Armonk, NY, USA) and R version 4.3.1. Categorical variables were summarised as counts (n) and percentages (%). Comparisons of women’s and men’s representation in session chair and speaker roles were conducted using Pearson’s chi-square test when expected cell frequencies were adequate; when expected cell frequencies were low, Fisher’s exact test was applied.

To assess temporal patterns in gender representation, logistic regression analyses were performed separately for session chairs, speakers, and all congress roles combined, with congress year entered as a continuous covariate and gender category (women vs. men) as the dependent variable. These models were used to evaluate whether the proportion of women changed over time, rather than to infer causal relationships or individual-level probabilities.

Subspecialty-specific logistic regression analyses were also performed to compare gender representation across subspecialties, with intensive care sessions as the reference category. Odds ratios (ORs) with 95% confidence intervals (CIs) were reported as measures of relative representation within the analysed congress roles.

All statistical tests were two-sided, and a *P* value <0.05 was considered statistically significant.

## Results

A total of 2,627 congress roles were analysed across TARK congresses held between 2015 and 2024, including 966 session chair roles and 1,661 speaker roles. Because the analysis was role-based, individuals who appeared in more than one session or role during the study period were counted separately for each congress role. The proportions of women and men were similar in both roles. Women accounted for 50.3% of session chairs (486/966), while men accounted for 49.7% (480/966). Similarly, among speakers, women represented 50.8% (843/1,661) and men 49.2% (818/1,661) of all roles (Table 1).

**Figure 1:**
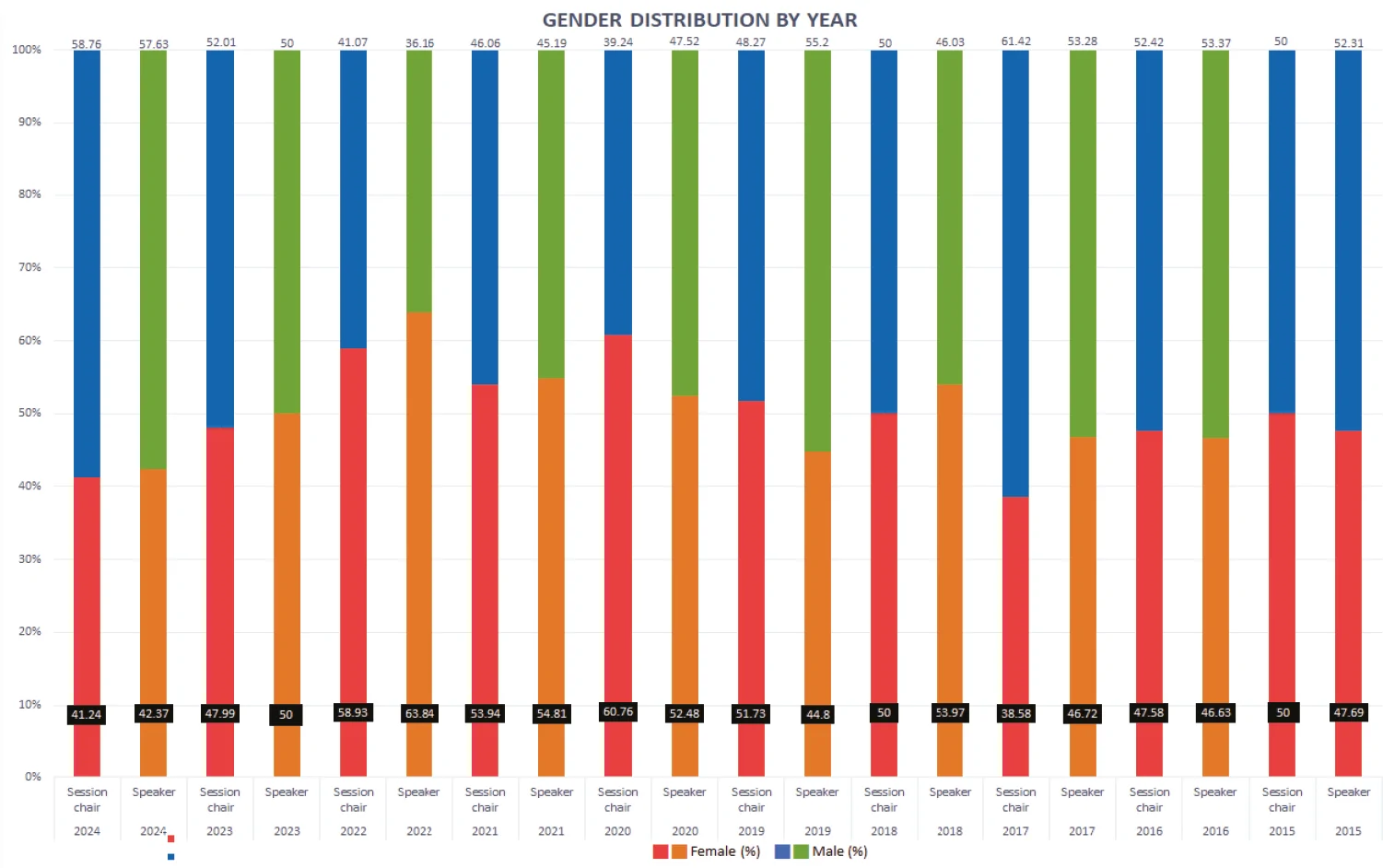
Annual distribution of women and men among session chairs and speakers at TARK Congresses (2015-2024). Bar charts present the annual proportions and absolute numbers of women and men serving as session chairs and speakers across TARK congresses between 2015 and 2024. The figure demonstrates year-to-year fluctuation without a consistent temporal increase or decrease in women’s proportional representation. TARK, Turkish Society of Anaesthesiology and Reanimation.

Figure 1 illustrates the annual distribution of women and men among session chairs and speakers. Across the study period, women’s representation fluctuated between approximately 30% and 60% in both roles, without a consistent upward or downward pattern. This descriptive pattern was consistent with the logistic regression results, which showed no statistically significant temporal change in women’s proportional representation.

Logistic regression analysis was performed to evaluate changes over time in women’s proportional representation (Table 2). For each one-year increase, the proportion of women did not change significantly. The OR was 1.01 for session chairs (95% CI: 0.96-1.05; *P*=0.71), 1.02 for speakers (95% CI: 0.99-1.06; *P*=0.17), and 1.01 when all roles were analysed together (95% CI: 0.98-1.04; *P*=0.51). These findings indicate that women’s representation did not show a statistically significant change over the study period.

Within-subspecialty analyses revealed variation in the distribution of women and men speakers (Table 3). In intensive care sessions, the proportion of male speakers was markedly higher, at 69.0%. In contrast, women represented 82.1% of speakers in obstetric and paediatric anaesthesia sessions, 62.5% in neuroanaesthesia, and 55.6% in cardiothoracic anaesthesia. Within-category comparisons revealed statistically significant gender differences across intensive care, neuroanaesthesia, and obstetric and paediatric anaesthesia sessions (Table 3).

In subspecialty-specific logistic regression analyses comparing women’s representation among speakers across subspecialties (Table 4), intensive care was used as the reference category. The highest proportional representation of women speakers was observed in obstetric/paediatric anaesthesia sessions (OR: 10.24; 95% CI: 5.09-20.60; *P*<0.001). Neuroanaesthesia (OR: 3.72; *P*<0.01) and cardiothoracic anaesthesia (OR: 2.79; *P*<0.01) were also associated with a significantly higher relative representation of women speakers compared with intensive care. Women’s representation among speakers was also higher in pain medicine and regional anaesthesia sessions than in intensive care sessions, although the magnitude of these differences was smaller than that observed for obstetric/paediatric anaesthesia, neuroanaesthesia, and cardiothoracic anaesthesia.

Leadership data from the TARK for the period 2014-2025 were evaluated descriptively (Table 5). The gender composition of society presidents and executive board members varied across terms, with some periods showing greater representation of women and others greater representation of men in specific roles. Because these data were summarised at the term level and not linked to individual speaker- or session-chair selection decisions, no causal or directional inferences were made regarding the relationship between leadership composition and congress-role allocation.

## Discussion

This study demonstrates that gender representation among session chairs and speakers at TARK congresses between 2015 and 2024 was numerically balanced overall; however, no significant improvement in women’s proportional representation was observed over the study period. Similarly, gender representation has been evaluated through other indicators of academic visibility, including publication output and editorial leadership, thereby highlighting the broader relevance of representation beyond clinical performance. Across the ten-year period, women accounted for approximately 50% of both the 966 session chair roles and the 1,661 speaker roles. Subspecialty-level analyses revealed a higher proportion of men in intensive care sessions, whereas women were more frequently represented in obstetric/paediatric anaesthesia and neuroanaesthesia. The descriptive assessment of society’s leadership showed variation across terms without a persistent predominance of either gender.

Interpretation of these congress-level findings requires consideration of the relevant reference population. Ideally, the proportions of women and men in roles at congresses should be compared with the gender distribution of registered society members or practising anaesthesiologists in Türkiye during the corresponding years. However, such year-matched and role-specific denominator data were not available for the present analysis. The only available national reference identified in the literature was derived from anaesthesiology and reanimation specialists working in educational institutions in Türkiye, among whom women constituted 57.7% of physicians. Therefore, an approximately 50% representation observed among TARK speakers and session chairs should be interpreted as numerical balance within congress roles, but not as definitive evidence of equitable opportunity relative to the broader workforce.^8^

The lack of a clear upward trend in the proportion of women serving as speakers or session chairs at TARK congresses over a decade suggests structural stability rather than progressive change, despite numerical parity. This pattern may be related to the recurrent use of established academic networks, invitation practices, and organisational routines carried over from previous years. In addition, subspecialty clustering, in which women concentrate in certain fields while remaining underrepresented in others, may partially explain why overall proportions remain stable despite balanced totals. These findings indicate that numerical equality alone does not necessarily translate into dynamic improvements and underscore the importance of addressing not only aggregate ratios but also distributional patterns and continuity in congressional representation.

Importantly, the absence of a significant temporal increase should not be interpreted as indicating a lack of informative findings. The main contribution of this study is the identification of a distinct national pattern in which overall numerical balance coexists with persistent subspecialty-based clustering. This finding adds context to the international literature, where the underrepresentation of women among invited speakers has commonly been reported, and suggests that aggregate parity alone may obscure uneven distribution across academic domains.

Compared with international literature, the findings of this study reveal a noteworthy contrast. Numerous studies across disciplines and countries have reported persistent underrepresentation of women among conference speakers, with some meetings showing a marked predominance of male speakers.^9, 10, 11^ Such patterns have been observed even in specialties where women constitute a numerical majority, suggesting an association with broader structural gender inequities.^12^ The findings demonstrated near parity between women and men across congress roles, while also revealing clear subspecialty-based clustering.

In Canada, retrospective analyses have shown persistent underrepresentation of women speakers at anaesthesiology conferences, with many sessions continuing to feature exclusively male speakers.^4^ Similar concerns regarding reduced academic visibility and unequal representation have been reported across multiple specialties and conference settings.^11, 13^ In this context, the numerical balance observed at TARK congresses appears more favourable than the underrepresentation reported in several international studies. Nevertheless, the absence of a significant temporal increase in women’s proportional representation suggests that this balance may reflect a stable, rather than progressively improving, structure.

Data from meetings of the ASA, the CAS, and the Society of Cardiovascular Anesthesiologists (SCA) consistently indicate insufficient representation of women among invited speakers.^3, 4, 14^ The notably low proportion of women speakers reported at JSA meetings (17.9%)-lower than those reported for ASA, CAS, and SCA-highlights geographic variability in gender underrepresentation.^6^ Conversely, some conferences have reported a higher-than-expected representation of women in specific fields, such as cardiothoracic anaesthesia.^15^ The subspecialty-based findings require cautious interpretation. The lower proportion of women speakers in intensive care sessions and the higher proportion observed in obstetric/paediatric anaesthesia and neuroanaesthesia sessions may reflect an unequal distribution of academic visibility across subspecialty domains rather than a simple overall gender imbalance. However, these differences cannot be interpreted as direct evidence of discrimination or intentional exclusion. Several factors may contribute to such clustering, including subspecialty workforce composition, clinical workload patterns, mentorship opportunities, academic networks, prior visibility in specific fields, and sociocultural expectations influencing career pathways. Because the present study did not include individual-level data on subspecialty membership, academic rank, workload, family responsibilities, or invitation processes, the mechanisms underlying these patterns could not be directly examined. Therefore, the observed subspecialty variation should be interpreted as a signal for further monitoring and targeted evaluation rather than as a causal explanation of gender-based opportunity differences.

The gender composition of scientific programme committees, session chairs, and organising boards has been identified as an important determinant of speaker representation. Prior studies have shown that the presence of at least one woman serving as a session chair or coordinator increases the likelihood that women will be invited as speakers.^7^ Similarly, other reports indicate that inclusion of women in programme leadership roles is associated with greater representation of women speakers.^16, 17, 18^ TARK leadership composition between 2014 and 2025 was evaluated descriptively and found to vary across terms, with no persistent predominance of either gender. Although previous studies have suggested that gender diversity among programme leaders or session coordinators may be associated with higher representation of women speakers, the present dataset did not allow direct testing of this relationship at the level of individual invitation or allocation decisions. Therefore, leadership findings should be interpreted as contextual, descriptive information rather than as evidence of a causal association with speaker or session chair representation.

Women’s representation in medicine often declines at higher academic and leadership levels.^19^ Because invited lectureships and moderator roles may contribute to academic visibility, disparities at scientific meetings can have implications for long-term academic advancement.^20^ Previous studies from Canada and the United States have demonstrated persistent underrepresentation of women in anaesthesiology authorship, editorial boards, society leadership, and advanced academic ranks, suggesting the presence of broader structural barriers within academic medicine.^21, 22, 23, 24, 25^

Previous studies have also shown that although women may outnumber men in academic anaesthesiology positions, men continue to dominate departmental leadership roles, and women experience slower promotion rates and lower publication output.^8^ These disparities have been attributed to factors such as disproportionate family and cultural responsibilities, long working hours, limited mentorship, and gender-based bias. Additionally, women have been reported to be less frequently nominated as speakers than their male counterparts.^26^ The balanced representation observed at TARK congresses may reflect the relatively high proportion of women anaesthesiologists and their strong representation in academic anaesthesiology in Türkiye, making these congresses comparatively balanced in numerical gender representation at the national level.

From a practical perspective, these findings may inform future scientific programme planning. The scientific committee and the executive board may consider monitoring gender representation across congress roles, subspecialty areas, and high-visibility sessions annually. Transparent criteria for speaker and session-chair invitations, broader nomination pools, and attention to subspecialty-specific imbalances may help maintain balanced representation and avoid concentration in particular academic areas. In addition, encouraging the participation of early-career anaesthesiologists and ensuring diversity among programme committees may support more inclusive and sustainable academic visibility at future congresses.

An overall gender balance among session chairs and speakers at TARK congresses over a ten-year period represents a notable departure from the underrepresentation of women commonly reported in the international literature. Nevertheless, pronounced subspecialty clustering and persistent gender disparities in academic leadership at both national and international levels indicate that representation patterns remain uneven across subspecialties and academic leadership domains. Maintaining visibility across gender groups, preserving balanced representation within scientific and organising committees, and supporting early-career anaesthesiologists across subspecialties may contribute to more inclusive academic visibility at future congresses.

### Study Limitations

This study has several limitations that should be acknowledged. First, the analysis was restricted to national TARK congresses and did not include other national or international anaesthesiology meetings; therefore, the findings may not be generalisable to all scientific meeting settings.

Second, self-identified gender information was not available in the congress programmes. Consequently, gender classification was performed using names, academic titles, congress documents, and publicly accessible institutional or professional information when clarification was required. Although a structured verification process was applied, the possibility of misclassification bias—particularly for gender-neutral names, international participants, or individuals with limited publicly available information—cannot be completely excluded. Gender representation was analysed within a binary women/men framework; non-binary gender identities could not be evaluated.

Third, year-matched denominator data for all practising anaesthesiologists or registered society members in Türkiye were not available. Therefore, the study could not determine whether congress representation was proportional to the eligible workforce across years, academic ranks, or subspecialties; the observed representation ratios should be interpreted cautiously.

Fourth, detailed participant-level information, including academic rank, institutional affiliation, years of experience, subspecialty workforce composition, workload characteristics, mentorship exposure, and invitation processes was not available. Accordingly, the study could not directly evaluate the mechanisms underlying subspecialty clustering patterns or representation differences.

Finally, because of the study’s retrospective and descriptive design, the findings should not be interpreted as evidence of causal relationships between leadership composition, congress organisation, and speaker or session chair representation.

## Conclusion

Gender representation among speakers and session chairs at the TARK congresses between 2015 and 2024 was numerically balanced overall, differing from the marked underrepresentation of women reported in many international anaesthesiology meetings. However, the absence of significant temporal improvement and the persistence of subspecialty-based clustering suggest that balanced overall representation does not necessarily indicate uniform distribution across all academic areas.

Because denominator data for the broader anaesthesiology workforce were unavailable, the findings should be interpreted as congress-level patterns of representation rather than as definitive evidence of equity of opportunity. Continued monitoring of representation trends, attention to subspecialty-specific imbalances, and inclusive scientific programme planning may support balanced academic visibility at future congresses.

## Ethics

**Ethics Committee Approval:** Ethical approval for this study was obtained from the Non-Interventional Research Ethics Committee of the University of Health Sciences Türkiye, Elazığ Fethi Sekin City Hospital (approval no: 2026/25-10, date: 05.03.2026).

**Informed Consent:** Owing to the retrospective design, the requirement for written informed consent was waived.

## Figures and Tables

**Table 1. Overall Distribution of Women and Men Among Session Chairs and Speakers:** 

**Role**	**Total n**	**Women (%)**	**Men (%)**	***P*** **value**
Speakers	1661	843 (50.75%)	818 (49.24%)	0.54
Session chairs	966	486 (50.31%)	480 (49.68%)	0.83

Pearson’s chi-square test was used for group comparisons; *P*<0.05 was considered statistically significant

**Table 2. Temporal Change in Women’s Representation in TARK Congress Roles (2015-2024), Logistic Regression Analysis:** 

**Role**	**OR**	**95% CI**	***P*** **value**
Speakers	1.02	0.99-1.06	0.17
Session chairs	1.01	0.96-1.05	0.71
All roles	1.01	0.98-1.04	0.51

Each row presents the estimated OR for a one-year increase in the year variable, along with the 95% CI and *P *value

OR, odds ratio; CI, confidence interval; TARK, Turkish Society of Anaesthesiology and Reanimation

**Table 3. Distribution of Women and Men Speakers Within Anaesthesiology Subspecialties:** 

**Subspecialty**	**Total** **speakers n**	**Women** **speakers n (%)**	**Men** **speakers n (%)**	***P* value**
Intensive care	84	26 (30.95%)	58 (69.04%)	<0.001
Pain	106	48 (45.28%)	58 (54.71%)	0.33
Regional anaesthesia	80	38 (47.5%)	42 (52.5%)	0.65
Neuroanaesthesia	64	40 (62.5%)	24 (37.5%)	0.046
Cardiothoracic anaesthesia	81	45 (55.5%)	36 (44.4%)	0.32
Obstetric and paediatric anaesthesia	95	78 (82.1%)	17 (17.89%)	<0.001

Percentages indicate the distribution of women and men speakers within each subspecialty. *P* values indicate within-category comparisons of women and men speaker counts. A *P* value <0.05 was considered statistically significant

**Table 4. Relative Representation of Women Speakers by Subspecialty (Logistic Regression Analysis):** 

**Subspecialty**	**Total speakers n**	**Women speakers** **n (%)**	**Men speakers** **n (%)**	**OR**	**95% CI**	***P* value**
Intensive care	84	26 (30.95%)	58 (69.05%)	1 (ref.)	–	–
Pain	106	48 (45.28%)	58 (54.72%)	1.85	1.01-3.36	0.045
Regional anaesthesia	80	38 (47.50%)	42 (52.50%)	2.02	1.07-3.82	0.031
Neuroanaesthesia	64	40 (62.50%)	24 (37.50%)	3.72	1.87-7.38	<0.001
Cardiothoracic anaesthesia	81	45 (55.56%)	36 (44.44%)	2.79	1.47-5.27	0.002
Obstetric and paediatric anaesthesia	95	78 (82.11%)	17 (17.89%)	10.24	5.09-20.60	<0.001

ORs represent the relative representation of women speakers compared with intensive care sessions as the reference category. CI (95% CI) and *P* values are reported from the logistic regression model

OR, odds ratio; CI, confidence interval

**Table 5. Descriptive Gender Distribution of Society Leadership Roles in the Turkish Society of Anaesthesiology and Reanimation (2014-2025):** 

**Term**	**President** **n (W/M)**	**Secretary general** **n (W/M)**	**Treasurer** **n (W/M)**	**Board member** **n (W/M)**
12.03.2023-23.02.2025	1/1	1/0	0/1	2/3
21.03.2021-12.03.2023	1/1	1/0	1/0	3/2
25.03.2018-21.03.2021	1/1	1/0	0/1	4/1
27.03.2016-25.03.2018	1/1	0/1	1/0	1/4
16.03.2014-27.03.2016	3/0	0/1	0/1	0/4

For each term, the number of women and men serving in listed leadership roles is presented descriptively. These data were not linked to individual speaker or session chair allocation decisions and were not used for causal inference. Categorisation was based on the same gender-classification procedure used for congress roles

W, women; M, men

## References

[1] Carr PL, Raj A, Kaplan SE, Terrin N, Breeze JL, Freund KM (2018). Gender differences in academic medicine: retention, rank, and leadership comparisons from the national faculty survey.. Acad Med.

[2] Files JA, Mayer AP, Ko MG (2017). Speaker introductions at internal medicine grand rounds: forms of address reveal gender bias.. J Womens Health.

[3] Moeschler SM, Gali B, Goyal S (2019). Speaker gender representation at the American Society of Anesthesiology annual meeting: 2011-2016.. Anesth Analg.

[4] Lorello GR, Parmar A, Flexman AM (2020). Representation of women amongst speakers at the Canadian Anesthesiologists’ Society annual meeting: a retrospective analysis from 2007 to 2019.. Can J Anesth.

[5] Mehta S, Rose L, Cook D, Herridge M, Owais S, Metaxa V (2018). The speaker gender gap at critical care conferences.. Crit Care Med.

[6] Kinoshita M, Sakai Y, Tanaka K (2025). Gender representation among speakers at the Japanese Society of Anesthesiologists meetings: a retrospective analysis. Waqar U, ed.. PLOS ONE.

[7] Ghatan CE, Altamirano J, Fassiotto M (2019). Achieving speaker gender equity at the SIR annual scientific meeting: the effect of female session coordinators.. J Vasc Interv Radiol.

[8] Hancı V, Altuntaş Uzun G, Aksoy M (2021). H-index and bibliometric analysis of scientific production parameters of the assistant academic anesthesiology and reanimation specialist in educational institutions in Turkey.. J Acad Res Med.

[9] Casadevall A (2015). Achieving speaker gender equity at the American Society for microbiology general meeting.. mBio.

[10] Ford HL, Brick C, Blaufuss K, Dekens PS (2018). Gender inequity in speaking opportunities at the American Geophysical Union Fall Meeting.. Nat Commun.

[11] Johnson CS, Smith PK, Wang C (2017). Sage on the stage: women’s representation at an academic conference.. Pers Soc Psychol Bull.

[12] Isbell LA, Young TP, Harcourt AH (2012). Stag parties linger: continued gender bias in a female-rich scientific discipline. Lambert JE, ed.. PLoS ONE.

[13] Carr PL, Gunn CM, Kaplan SA, Raj A, Freund KM (2015). Inadequate progress for women in academic medicine: findings from the national faculty study.. J Womens Health.

[14] Shillcutt SK, Lorenzen KA (2020). Whose voices are heard? Speaker gender representation at the Society of Cardiovascular Anesthesiologists annual meeting.. J Cardiothorac Vasc Anesth.

[15] Pan S, Shillcutt S, Oakes D, Muehlschegel JD, Shore-Lesserson L, Rong LQ (2022). Gender of abstract presenters at the Annual Meetings of the Society of Cardiovascular Anesthesiologists and American Society of Anesthesiologists: 2016 to 2020.. J Cardiothorac Vasc Anesth.

[16] Jones TM, Fanson KV, Lanfear R, Symonds MR, Higgie M (2014). Gender differences in conference presentations: a consequence of self-selection?. PeerJ.

[17] Moghaddam B, Gur RE (2016). Women at the podium: ACNP strives to reach speaker gender equality at the annual meeting.. Neuropsychopharmacology.

[18] Tougas C, Valtanen R, Bajwa A, Beck JJ (2020). Gender of presenters at orthopaedic meetings reflects gender diversity of society membership.. J Orthop.

[19] Where are all the women deans? AAMC. Accessed January 15, 2026..

[20] Klein RS, Voskuhl R, Segal BM (2017). Speaking out about gender imbalance in invited speakers improves diversity.. Nat Immunol.

[21] Lorello GR, Parmar A, Flexman AM (2019). Representation of women on the editorial board of the Canadian Journal of Anesthesia: a retrospective analysis from 1954 to 2018.. Can J Anesth Can Anesth.

[22] Flexman AM, Parmar A, Lorello GR (2019). Representation of female authors in the Canadian Journal of Anesthesia: a retrospective analysis of articles between 1954 and 2017.. Can J Anesth Can Anesth.

[23] Miller J, Chuba E, Deiner S, DeMaria S, Katz D (2019). Trends in authorship in anesthesiology journals.. Anesth Analg.

[24] Lorello GR, Flexman AM (2019). 75 years of leadership in the Canadian Anesthesiologists’ Society: a gender analysis.. Can J Anesth Can Anesth.

[25] Pagel PS, Freed JK, Lien CA (2019). Gender differences in authorship in the Journal of Cardiothoracic and Vascular Anesthesia: a 28-year analysis of publications originating from the United States, 1990-2017.. J Cardiothorac Vasc Anesth.

[26] Patton EW, Griffith KA, Jones RD, Stewart A, Ubel PA, Jagsi R (2017). Differences in mentor-mentee sponsorship in male vs female recipients of national institutes of health grants.. JAMA Intern Med.

